# Comparative Genomic Analysis of Shrimp-Pathogenic *Vibrio parahaemolyticus* LC and Intraspecific Strains with Emphasis on Virulent Factors of Mobile Genetic Elements

**DOI:** 10.3390/microorganisms11112752

**Published:** 2023-11-11

**Authors:** Ming Xue, Qi Gao, Rui Yan, Lingping Liu, Ling Wang, Binyu Wen, Chongqing Wen

**Affiliations:** Fisheries College, Guangdong Ocean University, Zhanjiang 524088, China; xuemtc@163.com (M.X.);

**Keywords:** *Vibrio parahaemolyticus*, comparative genomics, mobile genetic element, virulence factor, shrimp, vibriosis

## Abstract

*Vibrio parahaemolyticus* exhibits severe pathogenicity in humans and animals worldwide. In this study, genome sequencing and comparative analyses were conducted for in-depth characterization of the virulence factor (VF) repertoire of *V. parahaemolyticus* strain LC, which presented significant virulence to shrimp *Litopenaeus vannamei*. Strain LC, harboring two circular chromosomes and three linear plasmids, demonstrated ≥98.14% average nucleotide identities with 31 publicly available *V. parahaemolyticus* genomes, including 13, 11, and 7 shrimp-, human-, and non-pathogenic strains, respectively. Phylogeny analysis based on dispensable genes of pan-genome clustered 11 out of 14 shrimp-pathogenic strains and 7 out of 11 clinical strains into two distinct clades, indicating the close association between host-specific pathogenicity and accessory genes. The VFDB database revealed that 150 VFs of LC were mainly associated with the secretion system, adherence, antiphagocytosis, chemotaxis, motility, and iron uptake, whereas no homologs of the typical pathogenic genes *pirA*, *pirB*, *tdh*, and *trh* were detected. Four genes, *mshB*, *wbfT*, *wbfU*, and *wbtI*, were identified in both types of pathogenic strains but were absent in non-pathogens. Notably, a unique cluster similar to Yen-Tc, which encodes an insecticidal toxin complex, and diverse toxin–antitoxin (TA) systems, were identified on the mobile genetic elements (MGEs) of LC. Conclusively, in addition to the common VFs, various unique MGE-borne VFs, including the Yen-Tc cluster, TA components, and multiple chromosome-encoded chitinase genes, may contribute to the full spectrum of LC virulence. Moreover, *V. parahaemolyticus* demonstrates host-specific virulence, which potentially drives the origin and spread of pathogenic factors.

## 1. Introduction

With the growing demand for high-quality proteins, penaeid shrimp are becoming increasingly popular, leading to the rapid expansion of shrimp aquaculture worldwide. However, diseases attributed to *Vibrio* bacteria, i.e., vibriosis, occur frequently in various modes of intensive shrimp cultivation, causing substantial economic losses to the shrimp industry [1]. Meanwhile, some *Vibrio* species, especially *Vibrio parahaemolyticus*, have been recognized as the main causative agents of human gastroenteritis, wound infections, and infectious diarrhea in coastal countries due to consumption of undercooked shrimp or other seafood, causing severe damage in immunocompromised individuals [2,3]. Therefore, the potentially pathogenic nature of *Vibrio* and its adverse impact on humans and animals continue to be a research hotspot. 

Although multiple pathogenic mechanisms have been reported, the full spectrum of *Vibrio* virulence remains unclear, and a precise control strategy for vibriosis in aquatic animals is lacking. Among the known pathogenic *Vibrio* species, *V. parahaemolyticus* is frequently implicated in shrimp vibriosis, and much effort has been undertaken to determine its virulence determinants [4,5]. Various pathogenic factors of *V. parahaemolyticus* have been well defined and include adhesion factors, hemolysins, iron uptake systems, secretion systems, proteases, and lipopolysaccharides. These factors enable *V. parahaemolyticus* to adhere, invade, survive, and persist successfully in rearing water and shrimp [6,7]. Additionally, the pore-forming hemolysins, namely, thermostable direct hemolysin (TDH) and TDH-related hemolysin (TRH), and type III secretion (T3SS) systems, which inject toxins into eukaryotic cells, have been reported as the primary virulence factors of *V. parahaemolyticus*. T3SS2 is found only in Kanagawa phenomenon-positive isolates and is associated with enterotoxicity, whereas T3SS1 is ubiquitous in *V. parahaemolyticus* and correlated with cytotoxic activity [7,8]. Moreover, *V. parahaemolyticus*, carrying two plasmid-borne toxin genes, *pirA* and *pirB*, has been reported as the main pathogen causing acute hepatopancreatic necrosis disease (AHPND), which is one of the most severe bacterial diseases reported so far in cultivated shrimp and has caused tremendous economic losses since 2009 [1,9]. However, AHPND accounts for only a small proportion of disease outbreaks during shrimp culture [1,10]. Therefore, diverse types of vibriosis warrant comprehensive investigation, as many unknown genes may contribute to the pathogenicity of vibrios.

Since the pandemic serotype O3:K6 of *V. parahaemolyticus* emerged in India in 1996, various clinical strains of *V. parahaemolyticus* have been isolated globally, and contamination of coastal waters with O3:K6 has been reported [3,6]. The emergence of new pathogenic clones may be attributed to horizontal gene transfer (HGT) via mobile genetic elements (MGEs) [11,12]. Ceccarelli et al. reported that the acquisition of new genetic material via HGT plays a pivotal role in shaping the *V. parahaemolyticus* genome, as the rapid emergence of non-O3:K6 serogroups carrying pandemic markers has been observed [6]. Many strains of *V. parahaemolyticus* isolated from the environment were found to trigger cytotoxicity and enterotoxicity, although they were negative for *tdh*, *trh*, and T3SS2 [13]. Therefore, environmental *V. parahaemolyticus* strains with unknown etiology might pose potential risks to humans and animals. Furthermore, the pathogenicity of this bacterium may be a strain characteristic rather than a species characteristic, as some strains of the same species can be highly virulent, whereas others are avirulent [14,15]. Le Roux and Blokesch suggested that the virulence of different *Vibrio* strains differs considerably, and strain pathogenicity is highly associated with habitat [16]. Although *V. parahaemolyticus* is an intractable pathogen associated with both human and shrimp vibriosis, the phylogenetic relationship between clinical, shrimp-pathogenic, and non-pathogenic *V. parahaemolyticus* strains remains unclear. In this study, genomic data on emerging pathogens were used to identify some unique virulence-associated determinants by comparing LC with closely related strains of *V. parahaemolyticus*. The findings of this study are expected to provide novel insights into the full pathogenic mechanism of this species.

## 2. Materials and Methods

### 2.1. Strain Isolation and Cultivation 

Strain LC was isolated in 2018 from the intestine of diseased shrimp (*Litopenaeus vannamei*), which originated from an AHPND-like outbreak pond located in Zhanjiang City, Guangdong Province, China. This strain was identified as a member of the genus *Vibrio* based on its phenotype on TCBS plates and 16S rRNA gene sequencing. The isolated strains were preserved as frozen cultures in 20% glycerol at −80 °C, and 2216E broth (peptone 5.0 g/L, yeast extract 1.0 g/L, FePO_4_ 0.01 g/L, dissolved in natural seawater, pH 7.6–7.8) or agar was used for routine culture. 

### 2.2. Infection of Shrimp with Strain LC

To evaluate the pathogenicity of LC, shrimp infection challenges were evaluated separately using the injection and immersion methods. For the injection method, a total of 120 healthy *L. vannamei* juveniles (3.5 ± 0.2 g) were classified into three groups with four replicates each. Shrimps in each replicate were placed in a 25 L food-grade polypropylene bucket containing 10 L of seawater. Subsequently, they were intramuscularly injected in the third abdominal segment with 20 μL of LC, which was grown overnight in 2216E and suspended in 0.85% NaCl after harvesting logarithmic cells. Each individual in the low- and high-dosage treatment groups briefly received 20 μL of 5 × 10^6^ and 5 × 10^7^ colony-forming units (CFU) mL^−1^ of LC, respectively, whereas the control group received only 20 μL of 0.85% NaCl. During infection, the shrimp were fed twice a day, and the seawater (salinity 28, pH 8.1, 28 ± 2 °C) was constantly aerated, with a replacement of 30% volume daily. For the immersion method, a total of 360 healthy 10 days-old postlarvae (PL10) of *L. vannamei* were classified into three groups with three replicates each. Each replicate of PL10 was randomly placed in a bucket containing 10 L of seawater (salinity 28, pH 8.2, 32.5 ± 1.0 °C). The additional levels of LC in the seawater were 2 × 10^6^ and 1 × 10^5^ CFU mL^−1^ for the high- and low-dosage treatment groups, respectively, whereas no LC was added for the control group. The survival and morbidity of the shrimp were observed and recorded every 6 h until 72 h and 96 h for the immersion and injection methods, respectively. 

### 2.3. Genome Sequencing and Functional Annotation

DNA extraction, genome sequencing, gene prediction, and functional annotation of the LC strain were performed as previously described [17]. The assembled contigs were subjected to a built-in plasmid database using data downloaded from NCBI (https://ftp.ncbi.nlm.nih.gov/refseq/release/plasmid/, accessed on 10 July 2023). Plasmids were identified when the alignment length was >20% of the total sequence length (<1 Mb). The complete genome sequence of LC has been deposited in GenBank, with the accession numbers listed in Table 1. Genomic islands (GIs) were identified using IslandViewer 4, and islands with a size of ≥10 kb were retained (http://www.pathogenomics.sfu.ca/islandviewer/, accessed on 15 July 2023) [18]. Prophages were predicted using the phage search tool PHAST (http://phaster.ca/, accessed on 18 July 2023), and only intact prophages (score > 90) were retained [19]. Integrative conjugative element (ICEs) were identified online using ICEfinder (https://bioinfo-mml.sjtu.edu.cn/ICEfinder/ICEfinder.html, accessed on 24 July 2023). 

### 2.4. Calculation of Average Nucleotide Identity 

A total of 31 known *V. parahaemolyticus* strains with complete genomes (not draft genomes) were downloaded from NCBI (ftp://ftp.ncbi.nlm.nih.gov/genomes/, accessed on 30 July 2023). Of these 31 strains, 13 caused diseases in shrimp (shrimp-pathogenic strains); 11 triggered illnesses in humans (clinical strains), including the pandemic prototypic strain RIMD2210633 causing gastroenteritis [20]; and 7 were non-pathogenic. Details of strain type, origin, replicons, genome size, GC content, gene number, and accession numbers are listed in Table 1. Pairwise average nucleotide identity (ANI) indices of the 32 strains, including LC, were calculated using FastANI [21]. The resulting matrix was clustered and visualized in R v4.1.0 using the pheatmap package (https://cran.r-project.org/web/packages/pheatmap/index.html, accessed on 4 August 2023). Complete genomes of the *V. alginolyticus* strains 17749 and 33787, and the *V. harveyi* strains QT520 and 33843, were downloaded from the NCBI database and used as references. Additionally, ANIs among the plasmids or GIs of different strains were calculated using FastANI after blastn analysis.

### 2.5. Analysis of Pangenome and Phylogeny

Following genome annotation, the core genome alignment of LC and the 31 intraspecific strains was obtained with standard settings (minimum blastp identity of 90%) using the gff3 files derived from Prokka annotation, and subjected to Roary v3.8.0 [22,23]. A phylogenetic tree, based on the core genome, was constructed via concatenation of 3600 single-copy core genes with RAxML v8.1.22, using the maximum likelihood algorithm. The phylogenetic tree, based on dispensable genes of the pan-genome, was visualized using MEGA-X [24], with the output file of accessory binary genes in Newick format from Roary v3.8.0.

### 2.6. Identification of Virulence Factors

Putative virulence factors of LC and 31 isolates were retrieved by aligning the pre-annotated complete genomes in GenBank format with the Virulence Factors of Pathogenic Bacteria Database (VFDB) [25]. Considering that a large number of open reading frames (ORFs) on various MGEs were assigned as hypothetical proteins with unknown functions when annotated using Prokka, these genes were further manually curated by subjecting the coding sequences (CDSs) to NCBI blastp (https://blast.ncbi.nlm.nih.gov/Blast.cgi, accessed on 8 August 2023) to verify their functions. During this process, some virulence-associated genes were distinctly identified. The secretion systems of the sequenced strains were predicted online, using TXSScan (https://galaxy.pasteur.fr/, accessed on 20 August 2023).

### 2.7. Comparison of Gene Contents of GIs and Plasmids

Based on the phylogenomic and ANI analyses, six strains were selected for genome comparison with LC. Among these, strain RIMD2210633 (abbr. 2210633) had the highest ANI with LC; strains 20130629002S01 (S01) and 20151116002-3 (02-3) were the two closest relatives of LC in the core genome tree; and strains 20140624012-1 (12-1), 20140722001-1 (01-1), and 20140829008-1 (08-1) had the closest phylogenies with LC in the pangenome tree. Circular images of the two chromosomes of these seven strains were visualized using the BLAST Ring Image Generator (BRIG) tool [26], with all identified GIs, ICE, and prophages of LC labeled on the outermost ring. The gene contents of the largest GI-5, plasmids p96 and p71 of LC, with syntenic regions in other strains, were plotted using the Easyfig v2.3 software [27], with an emphasis on virulence-associated genes. Additionally, the specific genes of strain LC were sorted using Roary v3.8.0, according to the orthologous families that existed in LC but were absent in the other 31 genomes. 

### 2.8. Statistical Analysis

Shrimp survival rates (%) and average genomic sizes of *V. parahaemolyticus* strains were presented as the mean ± SD. After completing the arcsine square root conversion of the survival rates, these data were subjected to one-way analysis of variance to determine statistical significance (*p* < 0.05), followed by Tukey’s HSD post-hoc test, if a significant difference was detected using the package agricolae in R v4.1.0.

## 3. Results

### 3.1. Shrimp Performance after Infection

**Figure 1:**
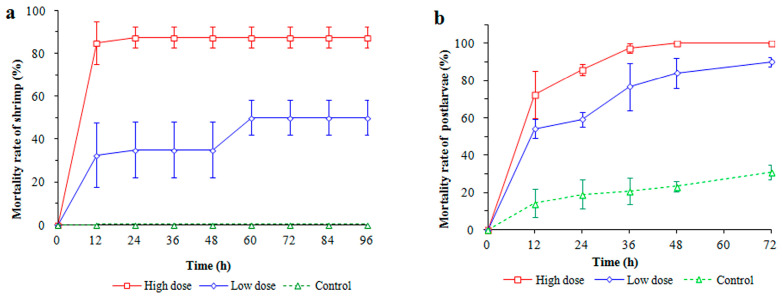
The mortality rates of *Litopenaeus vannamei* injected with low and high dosages of *V. parahaemolyticus* LC (**a**), and the mortality rates of *L. vannamei* postlarvae subjected to immersion infection with LC at low and high dosage (**b**).

In the injection challenge with LC at 48 h, shrimp mortalities were observed at 87.5% and 35.0% in the high- and low-dosage treatment groups, respectively. At 96 h, the mortality of shrimps in the low-dosage treatment rose to 50%, whereas no deaths occurred in the control group (Figure 1a). Shrimp mortalities in the high-dosage treatment were consistently significantly higher than those in the low-dosage treatment (*p* < 0.05). In the immersion test, at 12 h, the highest PL10 mortality was observed in the high-dosage treatment group (72.5%, *p* < 0.05) compared with those of the low-dosage treatment (54.2%) and control group (14.2%) (Figure 1b). Mortality continuously rose to 100% in the high-dosage treatment at 48 h. Overall, regardless of the infection mode, strain LC exhibited high virulence to shrimp, as mortalities in the LC-addition groups increased significantly compared with those in the control group. Moreover, LC-infected shrimp showed multiple disease symptoms, including broken appendages, a red tail, red intestine, red whiskers, an empty midgut, whitish muscle, and a red and swollen hepatopancreas. 

### 3.2. Genome Feature and ANI Analysis 

**Figure 2:**
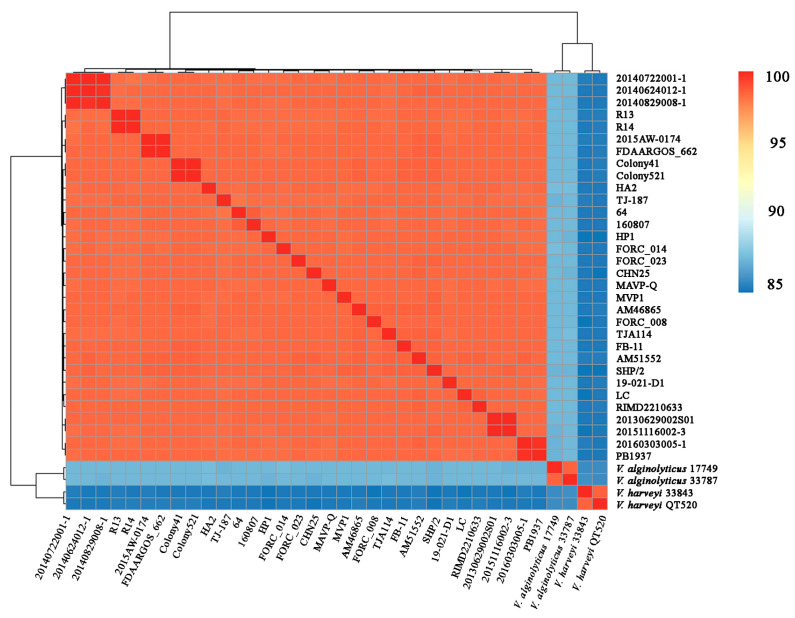
Average nucleotide identity analysis of genomes among *V. parahaemolyticus* LC with 31 intraspecific strains as well as two reference strains from *V. alginolyticus* and *V. harveyi*.

The genome size, G+C ratio, replicon, and gene number of strain LC are shown in Table 1. In total, 163 RNA genes were identified, including 34 rRNAs and 129 tRNAs. Of the 4979 predicted CDSs, 74.55% were assigned to COG functional categories. The sizes of the three plasmids, namely, p96, p71, and p60, were 96,124 bp, 70,872 bp, and 60,276 bp, respectively. Strain LC shared ≥98.14% ANIs with 31 other known *V. parahaemolyticus* strains, suggesting that LC belonged to *V. parahaemolyticus* and was closely related to the pandemic prototypic strain 2210633 (98.38% of ANI). Strain LC had ANIs of 83.84% and 86.15% for two closely related species, *V. harveyi* and *V. alginolyticus*, respectively (Figure 2), which were below the species threshold of 95–96% ANI. The average genomic size of the 14 shrimp-pathogenic strains (5.47 ± 0.20 Mb) was significantly larger (*p* < 0.05) than that of the 11 human-pathogenic stains (5.19 ± 0.13 Mb), whereas both did not present significant differences when compared with the seven non-pathogenic strains (5.37 ± 0.12 Mb). 

### 3.3. Phylogenome Analysis

**Figure 3:**
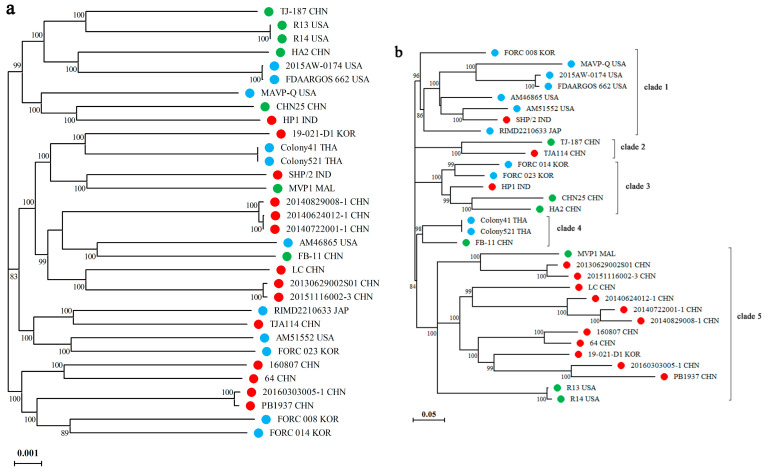
The phylogenomic trees were constructed based on the core genome of concatenated alignments of 3600 single-copy genes among 32 strains of *Vibrio parahaemolyticus* using the maximum likelihood method (**a**), and based on pangenome according to the presence/absence of 11,104 accessory genes using RAxML (**b**). Note: The scale bar indicates the number of substitutions per site (**a**) or the distance per 100 genes difference (**b**). Red circle: shrimp-pathogenic strains; blue circle: human-pathogenic strains; green circle: non-pathogenic strains. The abbreviations of CHN, USA, IND, MAL, KOR, JAP, and THA denote the strains originating from China, the United States, India, Malaysia, Korea, Japan, and Thailand, respectively.

Figure 3 shows the phylogenetic relationship of LC with 31 other *V. parahaemolyticus* strains based on the core and accessory genomes. As shown in Figure 3a, LC was closest to strains 20130629002S01(S01) and 20151116002-3 (02-3), both of which originated from Guangxi Province, China. Notably, five of the seven nonpathogenic strains clustered in the same clade, indicating a relatively close relationship between them. Overall, no apparent phylogenetic distinction was recognized regarding strain pathogenicity; similarly, a poor correlation was observed between strain phylogeny and the country of origin. Regarding the accessory genome, LC clustered most closely with the strains 20140624012-1 (12-1), 20140722001-1 (01-1), and 20140829008-1 (08-1), all of which originated from China (Figure 3b). Notably, 11 of the 14 shrimp-pathogenic strains resided in clade 5, of which almost all were derived from China, except for strain 19-021-D1, which originated from Korea, indicating the rapid dissemination of pathogenic strains off the coast of China. Except for SHP/2, the other seven strains in clade 1 were all human-pathogenic. This distinction indicates that accessory genes, associated with gain and loss, may play crucial roles in the pathogenicity of *V. parahaemolyticus*. Except for SHP/2, none of the other shrimp-pathogenic strains clustered together with isolates of human pathogenicity, whereas both clustered closely with non-virulent strains; therefore, different pathogenic strains may originate separately from environmental strains. 

### 3.4. Genome Comparison of LC with Six Closest Strains

**Figure 4:**
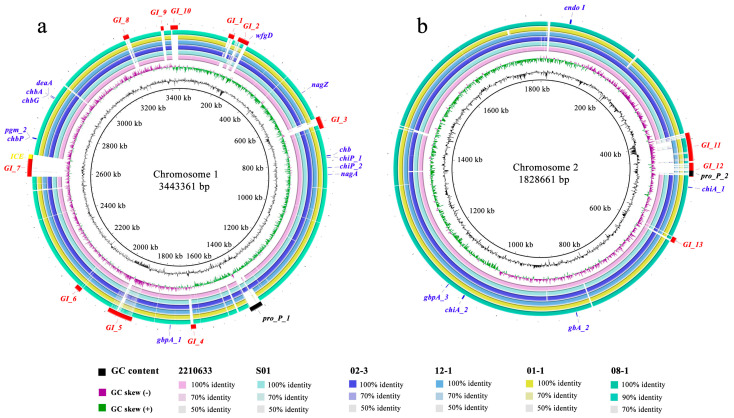
The circular comparison images of *V. parahaemolyticus* LC (central reference) with six closely related strains (2210633, S01, 02-3, 12-1, 01-1 and 08-1), regarding the large chromosome (**a**) and the small chromosome (**b**).

Figure 4 shows the comparisons of the large and small chromosomes of LC with those of strains 2210633, S01, 02-3, 12-1, 01-1, and 08-1. Overall, these seven strains share high nucleotide similarity, but there are also some variable regions, especially those that emerged in LC but were absent in the other six compared strains. In combination with the results derived from IslandViewer 4, PHAST, and ICEfinder, multiple MGEs, including 13 GIs, two prophages, and one ICE, are labeled on the LC genome. The names, sizes, G+C content, CDSs count, representative gene products, and potential functions of these MGEs are listed in Table S1. Among the GIs, GI-1 and GI-10 are primarily associated with resistance because they contain genes that encode proteins associated with resistance and immunity, such as S-type pyocin, colicin immunity protein, and immunity protein 49. GI-3, GI-5, and GI-7 are tentatively considered pathogenic GIs as they possess genes involved in multiple toxins and secretion system components. Other GIs, two prophages, and one ICE are mainly associated with metabolic functions because multiple genes encode proteins associated with metabolic properties. In addition, a total of 17 chitin-utilization genes are labeled on the LC genome, with 12 on the large chromosome and five on the small chromosome. Some genes emerge in multiple copies, such as two *chiA* homologs and three *gbpA* homologs. 

### 3.5. General Virulence Factors

A total of 150 virulence factors (VFs) were retrieved when the LC genome was subjected to the VFDB database. The functions of these VFs were mainly associated with secretion systems, adherence, antiphagocytosis, chemotaxis, motility, as well as iron uptake. The detailed information on various VF classes is listed in Table S2. Of the three hemolysin genes, only *tlh* was predicted, whereas *tdh* and *trh* were absent, and the AHPND-causing genes, *pirA* and *pirB,* were not detected. After comparing VFs among the 32 strains, genes *vasB*, *vasE*, *vasG*, and *vasH*, associated with the T6SS, only emerged in some shrimp-pathogenic strains, whereas 20 genes were present only in clinical strains, including *ureB*, *ureG*, *tdh*, *vopA*, *vopC*, *vopL*, *vopT*, *bpsE*, *bpsF*, *vcrD2*, *vscC2*, *vscN2*, and eight T3SS2-related genes. In addition, four genes: *mshB*, *wbfT*, *wbfU*, and *wbtI*, were present in both types of pathogenic strains but were absent in non-virulent strains.

### 3.6. Virulent Factors Associated with MGEs

**Figure 5:**
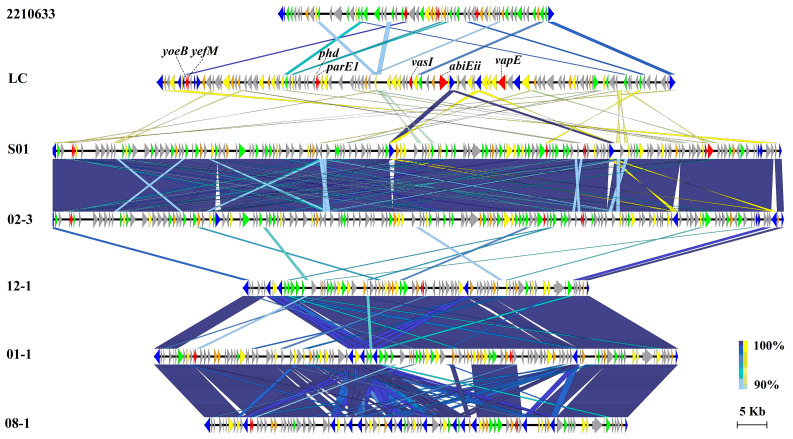
Comparison of the sequence identity and gene contents of GI-5 from *V. parahaemolyticus* LC with the syntenic regions from strains 2210633, S01, 02-3, 12-1, 01-1, and 08-1. Note: Each gene is displayed by a horizontal arrow in the direction of its coding strand (to scale), and color-coded using the designated key of the six classes, as follows: virulence factor (red), integrase/transposase/recombinase (blue), acetyltransferase domain/family (orange), other enzyme/domain (yellow), general functional gene (green), and hypothetical protein (light gray).

Of the 13 GIs possessed by LC, GI-5 was the largest, with a size of 93.2 kb. A comparison of GI-5 of LC with other bacterial genomes revealed some syntenic but highly variable regions (Figure 4). Overall, GI-5 of LC shared 87.1–88.3% ANIs with those of the other six strains, with several genes presenting ≥90% sequence identity to strains 2210633 and S01. The syntenic regions of GI-5 in some strains were extremely conserved, with sequence identities of up to 97.7–99.6% among 12-1, 01-1, and 08-1, and up to 99.9% between S01 and 02-3. Figure 5 shows the gene contents in these regions in order. These genes were primarily classified into six types based on their function annotation: mobility genes, virulence factors, general functional genes, acetyltransferase domain/family (GNAT), hypothetical proteins, and other enzymes/domains. Detailed information about the gene locus, gene name, and annotated functions are listed in Table S3.

Out of the 109 genes encoded on GI-5, 56 were specific to LC compared to the other 31 strains, and 31 of the 64 genes in the syntenic region of 2210633 were also specific to this strain, indicating the crucial role of GI-5 in the acquisition of alien genes. Moreover, multiple putative toxicity genes were detected in GI-5, including multiple type II/IV toxin-antitoxin (TA) systems, such as YefM/YoeB, Phd/YefM, parD/parE, and Txe/YoeB. In addition, other virulence factors reside on GI-5, such as the type VI secretion system VasI, EvfG, VC_A0118, and the virulence-associated E family protein. 

**Figure 6:**
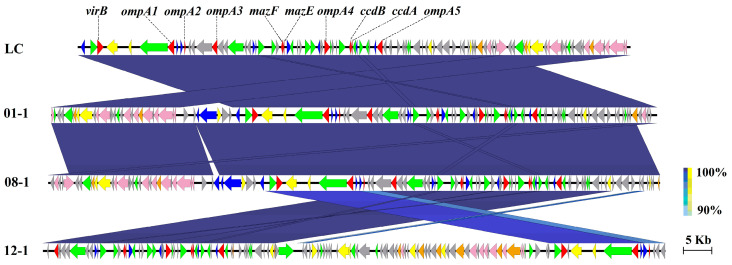
Comparison of the sequence identity and gene contents of p96 from *V. parahaemolyticus* LC with plasmids from strains 01-1, 08-1, and 12-1. Note: Each gene is displayed by a horizontal arrow in the direction of its coding strand (to scale) and color-coded using the designated key of the seven classes, as follows: virulence factor (red); integrase/transposase/recombinase (blue), conjugative transfer component (light pink), type II/III/IV secretion system (orange), hypothetical protein (light grey), other enzyme/domain (yellow); general functional gene/protein (green).

Comparison of p96 of LC with the other six bacterial genomes revealed that strains 01-1, 08-1, and 12-1 harbored highly similar plasmids with a similarity of 97.6–99.9% ANIs, especially the almost identical ones between 01-1 and 08-1 (Figure 6). Notably, ten virulence factors, including *mazE*, *mazF*, *virB*, ccdA, *ccdB,* and five copies of *ompA,* were detected on p96, and multiple genes associated with type III, IV, and VI secretion systems and their effectors were identified. BLAST analysis showed that these ten genes had homologs on plasmids of pathogenic strains 01-1, 08-1, and 12-1 (Table S4), suggesting that plasmid p96 might function as a carrier of virulence factors and facilitate the spread of virulent genes via conjugation transfer. 

**Figure 7:**
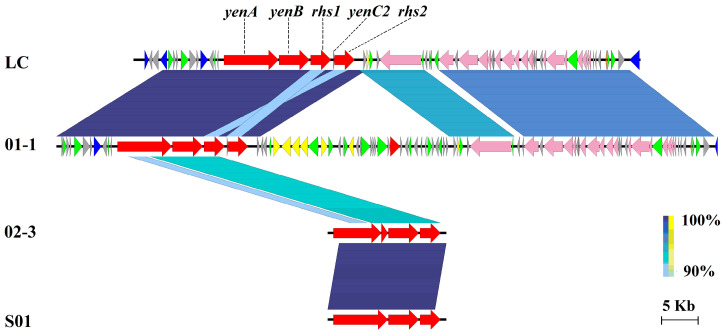
Comparison of the sequence identity and gene contents of p71 from *V. parahaemolyticus* LC with plasmid from strain 01-1, and with the chromosome-borne Tc cluster from strains 02-3 and S01. Note: Each gene is displayed by a horizontal arrow in the direction of its coding strand (to scale) and color-coded using the designated key of six classes, as follows: toxin complex genes (red), integrase/transposase/recombinase (blue), conjugative transfer component (light pink), other enzyme/domain (yellow), general functional gene (green), hypothetical protein (light grey).

As for p71 of LC, multiple conjugative transfer components were encoded on this plasmid, and a unique cluster comprised four genes: *yenA*, *yenB*, *Rhs*, and *yenC2*, which encode the toxin complex (Yen-Tc) subunit YenA, subunit YenB, rearrangement hotspot (RHS) repeat protein, and subunit YenC2, respectively. Strain 01-1 also harbored a similar plasmid with 98.1% ANI to p71 of LC, and the Yen-Tc cluster resided on this plasmid in the same order (Figure 7). Furthermore, two highly similar clusters of chromosomally encoded genes were also retrieved from strains 02-3 and S01, although they showed slightly different nucleotide sequences (Table S5). Accordingly, p71 of LC may also be a virulent MGE for *V. parahaemolyticus*, as Yen-Tc is considered an insecticidal agent. 

## 4. Discussion

In the present study, the topologies of the two phylogenomic trees differed considerably among the 32 *V. parahaemolyticus* strains. Based on the core genome, we observed a poor correlation between strain phylogeny and country of origin or pathogenic type. For example, strains FORC_023, AM51552, TJA114, and 2210633 resided in a subclade but came from Korea, the USA, China, and Japan, respectively. Similar to the results reported for *V. harveyi* and *V. alginolyticus* [17,28], the transcontinental spread of *V. parahaemolyticus* through the seafood trade or human activity also occurs frequently. In contrast, there was a relatively clear divergence in the two kinds of pathogenic strains regarding the accessory genome, i.e., 11 out of the 14 shrimp-pathogenic strains and seven of the 11 human-pathogenic strains separately clustered together, suggesting that the pathogenicity of *V. parahemolyticus* is closely associated with dispensable genes. Le Roux and Blokesch reported that vibrios can be divided into different phylogenetic clades based on habitat preferences, where habitats denote free-living/planktonic, particles, or organisms of small, medium, or larger size [16]. In this study, humans and shrimp may act as habitats for strains in clade 1 and clade 5, respectively. At the same time, the shrimp-pathogenic strains possessed significantly larger genome sizes than those of clinical isolates, implying that inhabitation may affect the genetic structure of *V. parahemolyticus* and more HGTs occurred among strains of the same host. 

Among the 11 shrimp-pathogenic strains in clade 5, 10 strains came from China, and another isolate, 19-021-D1, was from Korea, which is close to China. Moreover, LC clustered most closely with strains 08-1, 01-1, and 12-1 in the two phylogenomic trees, thus denoted that LC may originate from these phylogenetically coherent virulent strains through horizontal gene transfer or clonal expansion. Remarkably, clinical strains almost did not cluster together with shrimp-pathogenic ones, whereas these two kinds of strains readily clustered with non-virulent isolates. This may indicate distinct origins of the two types of pathogenic strains and a high relevance of host specificity with the pathogenicity of *V. parahaemolyticus*. 

Of the 248 specific genes in LC, 82.3% were associated with MGEs. For example, 56 specific genes resided on GI-5, indicating that novel genes were acquired mainly via HGT. Meanwhile, multiple ORFs on GI-5 were assigned as mobility genes, including integrase, transposase, and recombinase. There were seven mobility genes embedded in LC, and 14 distributed in the syntenic region of strain 08-1, thus confirming the high transferability of this island among *V. parahaemolyticus* strains, which facilitates genomic plasticity and the evolution of *V. parahaemolyticus* [6]. Similar to the other six intraspecific strains, a variety of genes encoding GNATs (domains/families) were scattered throughout GI-5 of LC. In bacteria, GNATs can act as aminoglycoside-passivating enzymes and are resistant to drugs, such as gentamicin, amikacin, and canamycin. Kazakov et al. reported that *Escherichia coli* acetyltransferase RimL could provide a basal level of resistance to antibiotics, microcin C, and albomycin via ribosomal protein acetylation [29]. The presence of a high frequency of GNAT-coding genes on one genomic island in vibrios is notable, and the physiological functions of GNATs in *V. parahemolyticus* warrant further investigation. 

Several bacterial TA systems have been identified in many pathogens, such as *E. coli*, *Streptococcus pneumoniae,* and *Staphylococcus aureus* [30,31]. Many reports have shown that TA systems are strongly associated with bacterial persistence, ensuring the safety and stability of MGEs, inducing the formation of persistent cells, and participating in biofilm formation, stress responses, antibiotic resistance, and host infection/pathogenicity [32,33,34]. However, relatively little information is known about the existence and role of TA systems in aquatic pathogenic bacteria, especially *Vibrio*, although Ma et al. reported that *YefM*-*YoeB* is involved in the response to adverse circumstances and the pathogenicity of *Edwardsiella piscicida* [33]. In the present study, multiple TA system components, including *YefM*, *ParE*, *AbiEii*, *AbiEi*, *mazF*, *mazE*, and *Txe*/*YoeB*, were detected in MGEs of GI-5, GI-3, and p96. Moreover, some homologs of these TA systems were also found in other strains. For example, *YefM* was identified in strains 2210633, 12-1, 01-1, and 08-1, and *ParE* was retrieved from S01, 02-3, 12-1, 08-1, and 01-1. Additionally, the antitoxin gene *RelB* was widely distributed in strains 2210633, S01, 02-3, 08-1, and 01-1, all of which demonstrate that various TA systems have been widely transmitted to *V. parahemolyticus*. Dy et al. reported that the *AbiEi*-*AbiEii* TA system is effective against phage infection, killing infected cells to protect most other cells [35]. Równicki et al. suggested that one of the important reasons for the emergence of antibiotic-resistant bacteria is the widespread distribution of TA systems in bacteria [32]. Considering the multiple TA systems distributed in the MGEs of LC, further work is needed to explore whether the presence of these TA systems provides competitive advantages for *V. parahemolyticus* in aquaculture settings.

Among three plasmids of LC, p60 presented extremely high ANIs of ≥99.1% with those of five strains S01, 02-3, 01-1, 08-1, and 12-1. As the majority of ORFs were assigned as hypothetical proteins, the function of p60 is entirely unclear, although it may be a conjugative plasmid containing several genes associated with the T4SS. As the largest LC plasmid, p96 possesses multiple virulence factors, including five homologs of *ompA*. Generally, *ompA* has been identified in many gram-negative bacteria that are pathogenic to humans and animals via adhesion and invasion during infection [36,37]. Bunpa et al. demonstrated that the OmpA protein is required for the pathogenicity of *V. alginolyticus,* as mutant bacteria had a significantly greater larval survival percentage of *Galleria mellonella* than those injected with the wild-type strain. Mutation of the *ompA* gene was strongly involved in the attenuation of the swarming ability, biofilm formation, and pathogenicity of *V. alginolyticus* to *G. mellonella* [38]. Homologs corresponding to the ten virulence factors in p96 were also found in strains 01-1, 08-1, and 12-1. Thus, p96 is a virulent plasmid with a high frequency of transferability. 

An interesting trait of strain LC is its possession of a unique cluster encoding a toxin complex family comprising subunit A, subunit YenB, subunit YenC2, and an RHS repeat protein. ABC toxins have been reported as high-molecular-weight protein complexes with insecticidal activity and were originally identified in the nematode-associated bacterium *Photorhabdus luminescens* [39]. This multi-subunit insecticidal ABC toxin complex was also found in *Yersinia entomophaga* (Yen-Tc), which typically contains three key proteins, designated A, B, and C, all of which are required for full toxicity [40]. Hurst et al. reported that Yen-Tc from *Y. entomophaga* displays an exceptionally virulent pathogenic phenotype in sensitive insect species, causing the death of insect larvae within 72 h of infection [41]. Moreover, this toxin complex is also a virulence factor of plant and human pathogens, such as *Salmonella enterica*, *Burkholderia pseudomallei*, *Pseudomonas syringae*, and *Pseudomonas fluorescens* [42]. However, there are few reports on this gene cluster in aquatic pathogens, except for a recent report on Tc toxins produced by *V. parahaemolyticus* [43]. 

In the present study, the Yen-Tc loci were not only plasmid-borne in strains LC and 01-1 but also chromosomally-encoded in strains of 02-3 and S01. This presence of Yen-Tc loci from the insect-pathogen *Y. entomophaga* in shrimp-pathogenic *V. parahemolyticus* strains provides evidence for genetic transfer, which is undoubtedly a major force in shaping the virulence of *V. parahaemolyticus*. The role of this complex in the shrimp mechanism of action remains unknown, and the LC-infected shrimp in this study showed signs of empty midgut, similar to pathognomonic AHPND lesions. However, the signs of whitish muscle and swollen hepatopancreas were unlike those of AHPND-diseased shrimp with pale-to-white and significant atrophy of the hepatopancreas [9,44], which may indicate different virulent mechanisms. The association of the Yen-Tc toxin with shrimp diseases warrants further exploration. 

Notably, a variety of chitin utilization genes were detected in strain LC, especially with multiple homologs of *chiA* and *gbpA*. Recently, some chitinases were reported to have more than one function beyond chitin degradation. Busby et al. suggested that several chitinases may be associated with toxin complexes [40]. Kehlet-Delgado et al. supported the hypothesis that chitin is a key mediator of interactions between oysters and *Vibrio* pathogens in view of the correlation between putative chitin utilization genes and pathogenicity [45]. Hurst et al. indicated that Yen-Tc was the first toxin complex to incorporate two chitinases as part of its structure for the full toxicity of *Y. entomophaga* MH96 to insect hosts [41]. Mahmood et al. reported that a novel insecticidal chitinase, ChiA, from the insect pathogen *Xenorhabdus nematophila*, was orally toxic to the crop pest, *Helicoverpa armigera* larvae, which showed a 9-fold reduction in weight, and the transformation of larvae into pupae was adversely affected when fed an artificial diet containing ChiA [46]. Therefore, chitinases may be multifunctional or specific to each homolog.

Chitin has been reported to facilitate HGT, not only by immediate transformation via induction of natural competence in *V. cholerae* [47], but also by T6SS through chitin-induced T6SS-mediated killing of neighboring nonkin cells, leading to the release of their genetic contents [16,48,49]. Considering the large amounts of chitin-containing molts and pellets in shrimp-rearing environments, the interaction of vibrios with shrimp via chitin may contribute to the high rate of HGT of pathogenic factors among different vibrios, which further exacerbates the vibriosis outbreak. From this perspective, many chitin-utilizing genes may also act as virulence factors in *V. parahemolyticus*. 

In this study, out of the 150 VFs of LC annotated by VFDB, only five were located on MGEs, including *pilW* on p71, and *wbfY*, *wbfV*, *wecA*, and *kpsF* on GI-2, which are associated with common bacterial virulence factors of adherence, antiphagocytosis, and colonization. However, the full virulence of LC is still far from clear, since a variety of novel ORFs on MGEs could not be functionally annotated using existing databases. For example, a total of 22 VFs, residing on GI-5, p96, and p71, were distinctly identified by NCBI blastp but not by VFDB. Meanwhile, these MGE-borne VFs may contribute significantly to the pathogenicity in shrimp via their frequent transferability. Regarding the VFDB-subjected VFs of all 32 strains, some genes could be indicative of strain pathogenicity since they are absent in nonvirulent strains. For instance, four genes: *vasB*, *vasE*, *vasG,* and *vasH*, associated with the T6SS, only emerged in certain shrimp-pathogenic strains. Conversely, *ureB*, *ureG*, *tdh*, *vopA*, *vopC*, *vopL*, *vopT*, *bpsE*, *bpsF*, *vcrD2*, *vscC2*, *vscN2,* and eight T3SS2-related genes were merely present in clinical isolates, while four genes: *mshB*, *wbfT*, *wbfU,* and *wbtI* existed in both kinds of pathogenic strains. Detailed information on the impacts of these genes from *V. parahemolyticus* on host shrimp needs further exploration.

## 5. Conclusions

In this study, besides known common virulence factors, some unique pathogenic factors, including multiple TA systems, chitin-utilization genes, and Yen-Tc complex, were identified in the genome of LC and some intraspecific strains. These factors may interact in an individual or synergistic manner, resulting in severe damage to shrimp. In particular, the impact of Yen-Tc on shrimp performance is of intrinsic interest for future research, because the isolated LC exhibited high virulence to shrimp but was armed with none of the typical pathogenic genes. Furthermore, in-depth investigations are needed in future studies to understand the actual effects of multiple chitinase genes in *V. parahemolyticus* on shrimp performance.

## Figures and Tables

**Table 1:** General information of strain LC and 31 other *V. parahaemolyticus* strains.

Strain Type	Strain Name (abbr.)	Countries of Origin	Genome (Mb)	G+C (%)	Replicons	No. Genes	Accession Numbers
Shrimp-pathogenic	LC	China	5.50	45.38	2 Chr., 3 Pla.	5142	CP119301-CP119305
	19-021-D1	Korea	5.58	45.35	2 Chr., 2 Pla.	5272	CP046411-CP046414
	160807	China	5.40	45.26	2 Chr., 1 Pla.	4990	CP033141-CP033143
	20151116002-3 (02-3)	China	5.46	45.24	2 Chr., 2 Pla.	5087	CP034305-CP034308
	20160303005-1	China	5.97	45.15	2 Chr., 5 Pla.	5662	CP034298-CP034304
	20140722001-1 (01-1)	China	5.56	45.27	2 Chr., 3 Pla.	5214	CP034289-CP034293
	20130629002S01 (S01)	China	5.40	45.36	2 Chr., 2 Pla.	5027	CP020034-CP020037
	20140829008-1 (08-1)	China	5.42	45.35	2 Chr., 2 Pla.	5061	CP034294-CP034297
	20140624012-1 (12-1)	China	5.35	45.32	2 Chr., 2 Pla.	4997	CP034285-CP034288
	HP1	India	5.08	45.31	2 Chr.	4644	CP069236-CP069237
	SHP/2	India	5.25	45.36	2 Chr., 2 Pla.	4817	CP066156-CP066159
	TJA114	China	5.57	45.20	2 Chr., 3 Pla.	5215	CP060087-CP060091
	PB1937	China	5.58	45.34	2 Chr., 2 Pla.	5361	CP022243-CP022246
	64	China	5.43	45.27	2 Chr., 2 Pla.	5305	CP074415-CP074418
Human-pathogenic	RIMD2210633 (2210633)	Japan	5.17	45.40	2 Chr.	4738	NC_004603, NC_004605
	FORC_008	Korea	5.04	45.44	2 Chr.	4661	CP009982-CP009983
	FORC_014	Korea	5.29	45.35	2 Chr., 1 Pla.	4943	CP011406-CP011408
	FORC_023	Korea	5.02	45.44	2 Chr.	4605	CP012950-CP012951
	Colony521	Thailand	5.39	46.17	2 Chr.	4452	CP078661-CP078661
	Colony41	Thailand	5.39	46.17	2 Chr.	4452	CP076383-CP076384
	FDAARGOS_662	USA	5.15	45.36	2 Chr.	4758	CP044070-CP044071
	2015AW-0174	USA	5.16	45.30	2 Chr.	4773	CP046753-CP046754
	MAVP-Q	USA	5.26	45.30	2 Chr.	4912	CP022472-CP022473
	AM46865	USA	5.09	45.47	2 Chr.	4712	CP046761-CP046762
	AM51552	USA	5.14	45.36	2 Chr.	4716	CP046759-CP046760
Non-pathogenic	R14	USA	5.44	45.27	2 Chr., 3 Pla.	5006	CP028141-CP028145
	R13	USA	5.44	45.27	2 Chr., 3 Pla.	4999	CP028342-CP028346
	MVP1	Malaysia	5.39	45.27	2 Chr., 1 Pla.	4945	CP043421-CP043423
	CHN25	China	5.44	45.19	2 Chr. 3 Pla.	5048	CP010883-CP010887
	HA2	China	5.28	44.93	2 Chr.	4869	CP023709-CP023710
	TJ-187	China	5.47	45.28	2 Chr., 3 Pla.	5149	CP068647-CP068651
	FB-11	China	5.13	45.40	2 Chr.	4709	CP073068-CP073069

Note: Chr. and Pla. indicate chromosome and plasmid, respectively.

## Data Availability

The datasets generated for this study can be found in the NCBI GenBank under accession numbers listed in Table 1.

## Supplementary Materials

The following supporting information can be downloaded at: https://www.mdpi.com/article/10.3390/microorganisms11112752/s1, Table S1: Features of GIs, prophages and ICE distributed on chromosome 1 and chromosome 2 of *Vibrio parahaemolyticus* LC; Table S2: The potential virulence factors of *Vibrio parahaemolyticus* LC retrieved from VFDB database; Table S3: Gene contents of GI-5 of *Virbrio parahaemolyticus* LC with homologs of other six intraspecific strains; Table S4: Gene contents of plasmid p96 of *Vibrio parahaemolyticus* LC with homologs of other three strains; Table S5: Gene contents of plasmid p71 of *Vibrio parahaemolyticus* LC with homologs of other three strains.
