# Supplementary material for: Comparative Genomic Analysis of Shrimp-Pathogenic Vibrio parahaemolyticus LC and Intraspecific Strains with Emphasis on Virulent Factors of Mobile Genetic Elements
Source: Microorganisms. 2023 Nov 11;11(11):2752. doi: 10.3390/microorganisms11112752 (PMC10672994; doi:10.3390/microorganisms11112752)
Supplement: Supplementary file 1 [file microorganisms-11-02752-s001.zip › Table S1.pdf]

Table S1 Features of GIs, prophages and ICE distributed on chromosome 1 and chromosome 2 of *Vibrio parahaemolyticus* LC

Replicon	Names	Size (Kb)	G+C (%)	No. of CDSs	Representative gene products	Potential function
Chromosome 1	GI-1	20.9	42.61	23	S-type pyocin; colicin immunity protein	Resistance
	GI-2	40.7	39.38	39	Bacterial sugar transferase; Polysaccharide biosynthesis protein	Metabolism
	GI-3	44.7	41.68	38	Putative AbiEii toxin; antitoxin component of TA system	Virulence
	GI-4	18.6	46.69	29	Cytochrome c oxidase subunit; sensor histidine kinase RcsC	Metabolism
	GI-5	93.2	40.33	113	Addiction module toxin, Txe/YoeB family; antitoxin YefM; ParE toxin, putative AbiEii toxin, Type VI; secretion system VasI, virulence-associated E family protein	Virulence and resistance
	GI-6	23.7	46.18	25	Isochorismatase family; FtsX-like permease family	Metabolism
	GI-7	64.6	46.24	67	Multiple proteins of conjugative transfer system	Virulence
	GI-8	20.6	43.04	23	Phage integrase family; inovirus Gp2	Metabolism
	GI-9	10.5	41.26	12	Serine recombinase PinR; phosphoglycolate phosphatase	Metabolism
	GI-10	27	43.93	28	Restriction-modification methylase; immunity protein 49	Resistance
	proP-1	48	41.7	64	DNA adenine methyltransferase YhdJ; Leucine efflux protein	Metabolism
	ICE	56.7	46.33	52	Aerobic cobaltochelatase subunit; anaerobic nitric oxide reductase transcription; cardiolipin synthase	Metabolism
Chromosome 2	GI-11	55.2	43.11	51	Endonuclease/phosphatase family; multiple proteins of conjugative transfer system	Metabolism
	GI-12	27.1	43.24	30	D12 class N6 adenine-specific DNA methyltransferase; guanosine monophosphate reductase	Metabolism
	GI-13	10.6	40.5	9	Adenosine deaminase; trypsin	Metabolism
	proP-2	10.7	40.94	11	Replicase family; phage regulatory protein CII	Metabolism
