# Supplementary material for: Comparative Genomic Analysis of Shrimp-Pathogenic Vibrio parahaemolyticus LC and Intraspecific Strains with Emphasis on Virulent Factors of Mobile Genetic Elements
Source: Microorganisms. 2023 Nov 11;11(11):2752. doi: 10.3390/microorganisms11112752 (PMC10672994; doi:10.3390/microorganisms11112752)
Supplement: Supplementary file 1 [file microorganisms-11-02752-s001.zip › Table S2.pdf]

Table S2 The potential virulence factors of *Vibrio parahaemolyticus* LC retrieved from VFDB database

VF classes	Virulence factors	Predicted genes	Gene loci	No. of genes
Adherence	MSHA type IV pilus	*mshA-mshN*	LC_02785--98	14
	Type IV pilus	*pilA--pilD, pilW*	LC_02586--89, LC_05018	5
Antiphagocytosis	Capsular polysaccharide	*cpsA--cpsJ*	LC_03830--39	10
		wbfV, wbfY, wecA	LC_00247, LC_00220, LC_00246	3
		wza, wzb, wzc	LC_04021, LC_04022, LC_04024	3
Chemotaxis and motility	Flagella	*cheA, cheB, cheR, cheV, cheW, cheY, cheZ*	LC_02208--09, LC_00761--62, LC_02205, LC_02210-11	7
		flaA, flaB, flaD, flaE, flaG, flaI	LC_02222, LC_02234, LC_02236--39,	6
		flgA--flgN	LC_00758--60, LC_00763--67, LC_00769--73	13
		flhA, flhB, flhF, flhG	LC_02213--16	4
		fliA, fliD--fliN, fliO--fliS	LC_02212, LC_02217--30, LC_02235, LC_02237	17
		flrA--flrC, motA-motB, motX--motY	LC_02231--33, LC_00659--60, LC_02109, LC_02897	7
Iron uptake	Enterobactin receptors	*irgA, vctA*	LC_02673, LC_04796	2
	Heme receptors	*hutA, hutR*	LC_03355, LC_03893	2
	ABC transport	*vctC, vctD, vctG, vctP*	LC_04789-92	4
Quorum sensing	Autoinducer-2	*luxS*	LC_02600	1
	Cholerae autoinducer-1	*cqsA*	LC_04842	1
Secretion system	EPS type II secretion system	*epsC, epsE--epsN, gspD*	LC_00125--36	12
	T3SS1 secreted effectors	*vopQ, vopR, vopS*	LC_01666, LC_01668, LC_01670	3
	T3SS1	*sycN, tyeA, vcrD, vcrG, vcrH, vcrR, vcrV*	LC_01651--52, LC_01644--48	7
		virF, virG, vopB, vopD, vopN, vxsC	LC_01684--86, LC_01642--43, LC_01653	6
		vscA--vscD, vscF--vscL, vscN, vscO--vscU, vscX--vscY	LC_01680--83, LC_01672--78, LC_01654--61,LC_01649--50	21
Toxin	Thermolabile hemolysin	*tlh*	LC_04391	1
Colonization and Immune evasion	Capsule biosynthesis and transport	*kpsF*	LC_00245	1
